# Prevention, Intervention and Care of Neurodegenerative Diseases

**DOI:** 10.3390/healthcare11162349

**Published:** 2023-08-21

**Authors:** Christos Bakirtzis, Marina-Kleopatra Boziki, Nikolaos Grigoriadis

**Affiliations:** Second Department of Neurology, Aristotle University of Thessaloniki, 54124 Thessaloniki, Greece; bozikim@auth.gr (M.-K.B.); ngrigoriadis@auth.gr (N.G.)

Chronic neurodegenerative diseases encompass a wide spectrum of disorders and affect millions of people worldwide. These diseases are characterized by progressive axonal loss that ultimately leads to apoptosis and consequently to neuronal death [1]. In the past years, neurodegenerative diseases have been extensively studied. We now understand that they may share common pathophysiological mechanisms, such as malfunctions in intracellular proteins [2,3], while the insidious underlying processes seen in these diseases often resemble those observed in the ageing brain [4]. Regardless of the progress which has been made until now, the feasibility of early detection and timely treatment of these diseases remains limited. This Special Issue gives voice to researchers who focus their attention on the efficient monitoring and treatment of neurodegenerative diseases.

Recent advances in the field of motor neuron diseases shed light to various underlying mechanisms implicated in their pathophysiology and revealed several useful biomarkers; nevertheless, no radical change has been observed in the clinical course of these diseases [5]. Although nowadays novel therapeutic approaches have been introduced in clinical practice, the need for non-pharmacological interventions remains unchanged. In this Special Issue, Paynter C. and colleagues investigated the impact of improving health literacy in people with motor neuron diseases [6]. For this study, 19 individuals with motor neuron diseases and 15 caregivers were recruited. According to the results, both patients and caregivers may benefit from personalized, individually adapted consultations and from the provision of information about ways to improve their daily living.

Similarly, non-pharmacological approaches may also ameliorate the quality of life of people living with other neurodegenerative conditions. One such approach is neurorehabilitation, which has proven to be a significant tool in the treatment of brain damage [7]. In this context, Hyun-Se Choi and Seung-Hyun Cho performed a randomized, multicenter study which included 60 patients with Parkinson’s disease [8]. They investigated whether a multimodal, patient-tailored approach in rehabilitation, combined with home modifications and caregiver education is superior to traditional rehabilitation techniques with regard to patients’ everyday living. They concluded that the proposed multimodal approach is more effective in improving the quality of life of both the patients and the family caregivers. Regarding the various approaches of neurorehabilitation, Garcia-Perez and colleagues explored the effectiveness of occupational therapies used in the rehabilitation of individuals with stroke [9]. They performed a systematic review including 13 relevant randomized controlled trials. According to the results, occupational therapy performed soon after hospital discharge combined with caregiver training is effective for neurological recovery. Furthermore, early intervention may reduce the cost of treatment and rehabilitation by improving patient adherence to the occupational therapeutic plan.

Dementia is one of the most common and detrimental manifestations of neurodegeneration. Extensive research focusing on the detection of biomarkers and potential risk factors for the development of dementia is being conducted [10]. Artificial intelligence and novel assessment tools may be valuable for the early detection and prognosis of dementia [11]. In this Special Issue, Aditya Shastry K. and colleagues examine the various deep learning approaches and techniques that have been developed for the detection of Alzheimer’s disease [12]. In their review, they focus on deep learning techniques of neuroimaging analyses and provide their strengths and limitations. Additionally, they address the various challenges that need to be overcome in order to use such technologies in everyday clinical practice. On the other hand, since people with cardiovascular diseases may be more prone to develop dementia, Buawangpong N and colleagues examined whether a cardiovascular risk score, widely used in Thailand, may help detect individuals at risk of developing mild cognitive impairment [13]. By reviewing the data of 421 participants, the authors concluded that the use of such scales for the quantification of cardiovascular risk factors may provide additional information about the risk of developing cognitive impairment, although further refinement of the tools used towards this direction is still needed.

Neurodegeneration is now at the center of focus of current research in multiple sclerosis and it is considered to be a major contributor in the accumulation of disability in these patients [14]. Therefore, disease monitoring may require the use of tools that potentially detect neurodegenerative processes early. Based on this, our study group discussed the use of optical coherence tomography and optical coherence tomography with angiography in multiple sclerosis [15]. In this review, we came up to the conclusion that the aforementioned studies of the retina may be useful in the early detection and monitoring of disease progression. The use of novel therapeutic agents that target cells involved in neurodegenerative processes, such as astrocytes and microglia, is under investigation [16,17]. However, until now, disease modifying treatments are mainly targeting lymphocytes. Ofatumumab, an anti-CD 20 monoclonal antibody, has been recently introduced in the therapeutic armamentarium of multiple sclerosis. In a brief report, Hafiza Munazza Taj and colleagues provide a critical appraisal of the four currently available randomized controlled studies that examine the efficacy of ofatumumab in relapsing multiple sclerosis, concluding that ofatumumab is an effective treatment for this type of the disease [18].

This Special Issue provides an interesting collection of articles focusing on early detection and management of various neurodegenerative diseases. We anticipate and we hope that in the near future, neurodegenerative diseases will be better understood and more efficiently treated. Nevertheless, further research is needed in order to achieve our goal which is to provide better care for our patients.
